# Distinguishing Zika and Dengue Viruses through Simple Clinical Assessment, Singapore 

**DOI:** 10.3201/eid2408.171883

**Published:** 2018-08

**Authors:** Gabriel Yan, Long Pang, Alex R. Cook, Hanley J. Ho, Mar Soe Win, Ai Leng Khoo, Joshua G.X. Wong, Chun Kiat Lee, Benedict Yan, Roland Jureen, Siew Seen Ho, David C. Lye, Paul A. Tambyah, Yee Sin Leo, Dale Fisher, Jolene Oon, Natasha Bagdasarian, Angela Chow, Nares Smitasin, Louis Yi Ann Chai

**Affiliations:** National University Health System, Singapore (G. Yan, L. Pang, A.R. Cook, M.S. Win, S.S. Ho, P.A. Tambyah, D. Fisher, J. Oon, N. Bagdasarian, N. Smitasin, L.Y.A. Chai);; National University of Singapore (L. Pang, A.R. Cook, P.A. Tambyah, D. Fisher, N. Bagdasarian, L.Y.A. Chai);; Tan Tock Seng Hospital, Singapore (H.J. Ho, J.G.X. Wong, D.C. Lye, Y.S. Leo, A. Chow);; National University Cancer Institute, Singapore (M.S. Win, L.Y.A. Chai);; National Healthcare Group, Singapore (A.L. Khoo);; National University Hospital, Singapore (C.K. Lee, B. Yan, R. Jureen)

**Keywords:** Southeast Asia, dengue virus, Zika virus, conjunctivitis, platelets, viruses, zoonoses, Singapore

## Abstract

Dengue virus and Zika virus coexist in tropical regions in Asia where healthcare resources are limited; differentiating the 2 viruses is challenging. We showed in a case–control discovery cohort, and replicated in a validation cohort, that the diagnostic indices of conjunctivitis, platelet count, and monocyte count reliably distinguished between these viruses.

Zika virus and dengue virus (DENV) are arboviral infections transmitted by the *Aedes* mosquito. Dengue is endemic in Singapore with >10,000 case notifications annually (*1*). Although Zika virus was known through serosurveys to circulate in Southeast Asia (*2*), confirmed infections had been scarce until August 2016, when the first recognized outbreak in Southeast Asia occurred in Singapore, following the epidemic in the Americas (*3*).

Co-circulation of both viruses poses challenges to healthcare providers in distinguishing between the 2 infections. These infections have similar clinical features, including fever, rash, and myalgia. Because most patients enter the primary healthcare setting with nonspecific symptoms, we sought to determine if either infection had distinguishing symptoms, signs, or basic laboratory findings.

## The Study

We conducted a case–control study at the National University Hospital with ethics approval from the hospital’s Institutional Review Board. Patients infected with Zika virus and DENV who were seen at the hospital in 2016 constituted the discovery cohort. We confirmed Zika virus infection through testing for viral RNA in serum or urine, as described by Lanciotti et al. (*4*). We confirmed DENV infection through testing for serum DENV nonstructural protein 1 (NS1) antigen (SD BIOLINE Dengue DUO Kit; Standard Diagnostics, Kyonggi-do, South Korea) or by reverse transcription PCR (*5*). The clinical information collected included demographics, symptomatology, examination findings, and laboratory investigations, including complete blood count (with the monocyte count automated) and liver function test.

We compared clinical characteristics of both infections by univariate logistic regression against dichotomous symptomatology and continuous laboratory parameters. We selected predictors that could differentiate Zika virus and DENV infection as input for subsequent multivariate regression models and computed the area under the receiver operating characteristic curve (AUC) to compare model performance. We validated the results in a separate cohort of Zika virus and DENV patients from Tan Tock Seng Hospital, Singapore (*5*). From this validation cohort, we ascertained AUC and accuracy of the derived predictors. There were no pregnant patients in either cohort. We performed all analyses with R statistical software version 3.3.1 (http://www.R-project.org).

We identified 121 patients for the discovery study; 34 had Zika virus and 87 had DENV infection. Fifteen Zika patients (44.1%) were male and 19 (55.9%) female; 57 (65.5%) DENV patients were male and 30 (34.5%) female. Thirty-one Zika patients (91.1%) were PCR positive by urine test and 3 (8.9%) by plasma.

**Figure 1:**
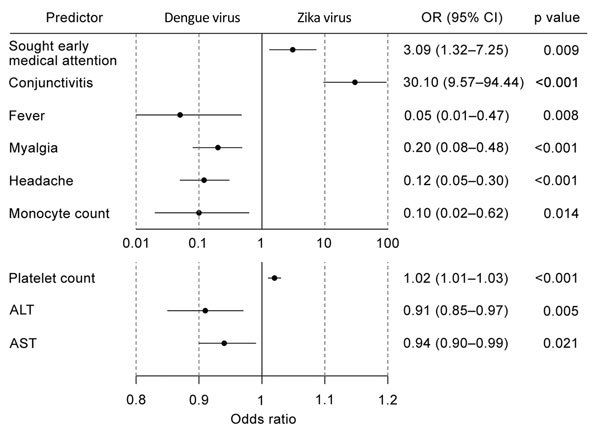
Univariate logistic regression model of clinical characteristics for patients in study of clinical assessments to distinguish Zika and dengue virus infections, Singapore. We analyzed early presentation (seeking treatment within 3 days of symptom onset), conjunctivitis, fever, myalgia, and headache as dichotomous variables, and laboratory findings (monocyte and platelet counts, ALT and AST levels) as continuous variables. For dichotomous variables, odds ratio (OR) >1 is predictive of Zika virus infection and <1 of dengue virus infection; for continuous variables, every unit increase in readout is predictive of Zika virus infection for OR >1 and dengue virus infection for OR <1. Error bars indicate 95% CIs. ALT, alanine aminotransferase; AST, aspartate aminotransferase.

Zika patients sought treatment earlier in their illness than did DENV patients. Whereas viral symptoms including fever and arthralgia were common to both, differences were discernible (Figure 1). Conjunctivitis strongly indicated Zika virus infection (odds ratio [OR] 30.1, 95% CI 9.57–94.44; p < 0.001). In contrast, fever (OR 0.05, 95% CI 0.01–0.47; p = 0.008), myalgia (OR 0.20, 95% CI 0.08–0.48; p<0.001), and headache (OR 0.12, 95% CI 0.05–0.30; p<0.001) were more prominent in patients with DENV infection.

Further, DENV patients tended to have thrombocytopenia (median platelet count 132 × 10^9^/µL, range 15–386 × 10^9^/µL) and monocytosis (median monocyte count 0.50 × 10^9^/µL, range 0.11–1.70 × 10^9^/µL), whereas Zika patients tended to have normal platelet (median 225 × 10^9^/µL, range 128–326 × 10^9^/µL; p<0.001) and monocyte (median 0.35 × 10^9^/µL, range 0.13–1.00 × 10^9^/µL; p = 0.021) counts. The odds of Zika virus infection increased 2% with every unit (10^9^/µL) increase in platelet count (OR 1.02, 95% CI 1.01–1.03; p<0.001) (Figure 1). Lower monocyte counts were associated with Zika virus infection (OR 0.10, 95% CI, 0.02–0.62; p = 0.014).

Patients with DENV had biochemical evidence of liver injury with hepatic alanine aminotransferase (ALT) and aspartate aminotransferase (AST) levels >2 times the upper reference limit (ALT, median 51.0, range 12–465 U/L; AST, median 65, range 20–720 U/L). The reference range for ALT is 10–70 U/L, and for AST, 10–50 U/L. In contrast, Zika virus patients did not have pronounced abnormalities in albumin, ALT, AST, or alkaline phosphatase levels.

**Figure 2:**
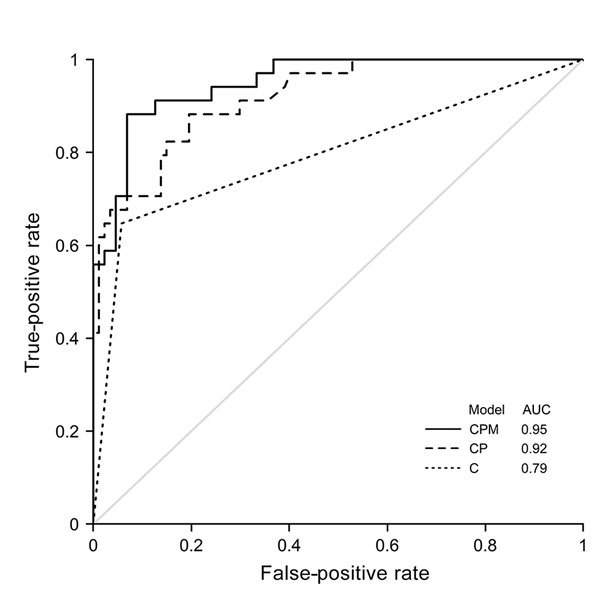
Receiver operating characteristics for different models in study of clinical assessments to distinguish Zika and dengue virus infections, Singapore. AUC is shown for different models: conjunctivitis alone (model C), conjunctivitis with platelet count (model CP), and conjunctivitis with platelet and monocyte counts (model CPM). AUC, area under the curve.

Our findings point to conjunctivitis, platelet, monocyte, ALT, and AST levels as candidate markers to differentiate Zika virus patients from DENV patients. Conjunctivitis alone had an AUC of 0.79 in identifying Zika virus patients; normal platelet count in addition to conjunctivitis increased the AUC to 0.92, and adding a normal monocyte count further improved the AUC to 0.95 (Figure 2). The use of these 3 indices (conjunctivitis and platelet and monocyte counts) had 88% sensitivity and 93% specificity in distinguishing Zika virus from DENV, with a diagnostic accuracy of 92%. Inclusion of ALT and AST, however, did not further enhance the diagnostic capability.

We applied these 3 indices to a validation cohort consisting of 25 Zika virus and 70 DENV patients (Table 1), resulting in an AUC of 0.90. Applying a cutoff score of 0.34 as determined by Youden’s index to maximize sensitivity and specificity of our original model to the new validation dataset, the positive predictive value was 83% and negative predictive value 87%, achieving a similar diagnostic accuracy of 86%.

**Table 1:** Patient profile for validation cohort in study of clinical assessments to distinguish Zika and dengue virus infections, Singapore

Characteristic	Value, N = 95
Age	Median 38, mean 37.9, range 21−67
Sex	M 72, F 23
Day of illness*	Median 5, mean 4.7, range 2−9
Conjunctivitis	Yes 13, no 82
Fever	Yes 93, no 2
Myalgia	Yes 33, no 62
Headache	Yes 33, no 62
Monocyte count, × 10^9^/µL	Median 0.32, mean 0.39, range 0.08−1.38
Platelet count, ×10^9^/µL	Median 99, mean 115.2, range 13−308
Alanine aminotransferase, U/L	Median 33, mean 55.2, range 12−677
Aspartate aminotransferase, U/L	Median 44, mean 76.5, range 17−715

*Day on which care was sought.

Zika virus and DENV coexist in many developing nations in equatorial South America and Southeast Asia, where there is limited accessibility to health resources and virus-specific diagnostics are not readily available. Differentiating Zika virus and DENV infections early is important in the prognostication and subsequent monitoring and follow-up of these patients. Although Zika virus infection is self-limiting, concerns about its sequelae in pregnant women and birth defects are well established (*6*). In contrast, severe DENV infection leads to debilitating illness that can cause vascular leakage, dengue shock, and death (*7*).

We applied both definitions from the US Centers for Disease Control and Prevention (CDC) and World Health Organization (WHO) for suspected Zika cases (*8*,*9*) in our patient cohort and found them to be unsatisfactory in distinguishing Zika virus from DENV patients (CDC, sensitivity 100%, specificity 2%; WHO, sensitivity 71%, specificity 67%) (Table 2). We therefore sought to develop more accurate indices to identify Zika virus among the backdrop of DENV cases in Singapore.

**Table 2:** Sensitivity and specificity using CDC and WHO definitions of suspected Zika virus infection in study of clinical assessments to distinguish Zika and dengue virus infections, Singapore*

Case definition			Characteristic					
Source	Criteria		Patient meets criteria	Zika virus positive, n = 34	Zika virus negative, n = 57	Total, n = 91	Sensitivity, %	Specificity, %
CDC	Clinically compatible illness with >1 of the following not explained by another etiology: fever, rash, arthralgia, or conjunctivitis†		Yes	34	56	90	100	2
WHO	Fever and/or rash and any of the following: arthralgia, arthritis, nonpurulent conjunctivitis		Yes	24	19	43	71	67
			No	10	38	48		

*CDC, Centers for Disease Control and Prevention; WHO, World Health Organization. †We excluded 2 additional criteria, complications of pregnancy and neurologic manifestations, because they were not present in our study population.

Our results highlight the utility of conjunctivitis and normal platelet and monocyte counts to distinguish Zika virus infection. We found conjunctivitis to be already a strong predictor of Zika virus infection. The study by Waggoner et al. in Nicaragua had reported conjunctivitis and rash in association with Zika virus infection (*10*). However, rash was not prominent among Zika patients in our study. Headache and myalgia were more common in DENV (*7*) and could help to distinguish DENV from Zika virus in our cohort. Prior studies had not ascertained if incorporation of basic laboratory indices could further enhance diagnostic capability. In our univariate logistic regression model, thrombocytopenia, transaminitis, and monocytosis were notable in DENV infection. Conversely, Zika patients tended to have normal platelet, aminotransaminase, and monocyte levels. 

## Conclusions

We were able to derive 3 simple clinical predictors on the basis of our findings: in the presence of conjunctivitis and normal platelet and monocyte counts, diagnostic AUC for Zika increased from 0.79 to 0.95, with 92% accuracy (88% sensitivity and 93% specificity). The accuracy of our derived indices exceeds that of WHO’s and CDC’s definitions for Zika case identification, notwithstanding that performance may differ with disease prevalence or population factors. Distinguishing Zika virus from DENV infection on clinical grounds remains daunting, and it will be ideal to validate these derived indices in a prospective patient cohort. Until then, these simple clinical assessments using conjunctivitis and basic blood count parameters will be helpful in regions of the world where both Zika virus and DENV are endemic.
